# Big data and data repurposing - using existing data to answer new questions in vascular dementia research

**DOI:** 10.1186/s12883-017-0841-2

**Published:** 2017-04-17

**Authors:** Fergus N. Doubal, Myzoon Ali, G. David Batty, Andreas Charidimou, Maria Eriksdotter, Martin Hofmann-Apitius, Yun-Hee Kim, Deborah A. Levine, Gillian Mead, Hermann A. M. Mucke, Craig W. Ritchie, Charlotte J. Roberts, Tom C. Russ, Robert Stewart, William Whiteley, Terence J. Quinn

**Affiliations:** 1 Stroke Association Garfield Weston Foundation Clinical Senior Lecturer, Centre for Clinical Brain Sciences, University of Edinburgh, Edinburgh, UK; 2 VISTA and VICCTA Coordinator, Institutes of Cardiovascular and Medical Sciences, University of Glasgow, Glasgow, UK; 3 Reader in Epidemiology, Department of Epidemiology & Public Health, University College London, London, UK; 4 J Philip Kistler Stroke Research Centre, Department of neurology, Massachusetts General Hospital Stroke Research Centre, Harvard medical School, Boston, MA USA; 5 Division of Clinical Geriatrics, Department of Neurobiology, Care Sciences and Society, Karolinska Institutet and department of Geriatric Medicine, Karolinska university hospital, Stockholm, Sweden; 6 Chair and Head of Department, Fraunhofer Institute for Algorithms and Scientific Computing, Schloss Birlinghoven, Sankt Augustin, Germany; 7 Department of Physical and Rehabilitation Medicine, Centre for Prevention and Rehabilitation, Heart Vascular and Stroke Institute, Samsung Medical Centre, Sungkyunkwan University School of Medicine, Seoul, Republic of Korea; 8 Department of Internal Medicine, University of Michigan and the VA Ann Arbor Healthcare System, Ann Arbor, MI USA; 9 Centre for Clinical Brain Sciences, University of Edinburgh, Edinburgh, UK; 10 Life Sciences Consultant, H.M. Pharma Consultancy, Wien, Austria; 11 Centre for Dementia Prevention, University of Edinburgh, Edinburgh, UK; 12 ICHOM International Consortium for Health Outcomes Measurement, Hamilton House, Mabledon Place, London, WC1H 9BB UK; 13 Marjorie MacBeath Intermediate Clinical Fellow, Alzheimer Scotland Dementia Research Centre, & Centre for Dementia Prevention, University of Edinburgh, Edinburgh, UK; 14 King’s College London (Institute of Psychiatry, Psychology and Neuroscience), South London and Maudsley NHS Foundation Trust, London, UK; 15 MRC Clinician Scientist and Honorary Consultant Neurologist, Centre for Clinical Brain Sciences, University of Edinburgh, Edinburgh, UK; 16 Institute of Cardiovascular and Medical Sciences, University of Glasgow, Glasgow, UK

**Keywords:** Big data, Data, Clinical Trials, Cohort studies, Dementia, Electronic health records, Systematic review, Registries, Vascular dementia

## Abstract

**Introduction:**

Traditional approaches to clinical research have, as yet, failed to provide effective treatments for vascular dementia (VaD). Novel approaches to collation and synthesis of data may allow for time and cost efficient hypothesis generating and testing. These approaches may have particular utility in helping us understand and treat a complex condition such as VaD.

**Methods:**

We present an overview of new uses for existing data to progress VaD research. The overview is the result of consultation with various stakeholders, focused literature review and learning from the group’s experience of successful approaches to data repurposing. In particular, we benefitted from the expert discussion and input of delegates at the 9^th^ International Congress on Vascular Dementia (Ljubljana, 16-18^th^ October 2015).

**Results:**

We agreed on key areas that could be of relevance to VaD research: systematic review of existing studies; individual patient level analyses of existing trials and cohorts and linking electronic health record data to other datasets. We illustrated each theme with a case-study of an existing project that has utilised this approach.

**Conclusions:**

There are many opportunities for the VaD research community to make better use of existing data. The volume of potentially available data is increasing and the opportunities for using these resources to progress the VaD research agenda are exciting. Of course, these approaches come with inherent limitations and biases, as bigger datasets are not necessarily better datasets and maintaining rigour and critical analysis will be key to optimising data use.

## Background

Traditional approaches to clinical research, such as the randomised controlled trial (RCT), have facilitated major advances in our understanding and treatment of common diseases. In the stroke field we now have robust evidence for many aspects of acute care [1]. In comparison evidence based interventions for prevention and treatment of vascular dementia (VaD) are relatively sparse [2].

Although there are few RCTs in VaD, data do exist. There are several alternative sources and approaches to data that could be used for time- and cost-efficient research. Exploiting novel methods of data collation and synthesis may allow us to develop the VaD evidence base, where traditional study designs have failed to deliver. Repurposing existing resources to allow for original research in VaD is aligned with current moves towards improving research efficiency and reducing waste [3], a theme of increasing importance to funders and peer reviewers.

In this overview we describe a variety of approaches to data, providing background and illustrate with case-studies. We will discuss a series of complementary research methodologies (Table 1). We do not claim that the overview is exhaustive and we recognise there is a degree of overlap. The overview will keep a VaD focus where possible, although the techniques discussed are applicable to many research areas. Some of the research approaches are relatively new and for some, as yet, there are no specific examples of their application in the VaD field. In these situations we take exemplars from other areas of research.

**Table 1:** Summary of methods and their potential strengths and weaknesses for data in VaD

Approach	Potential strengths	Potential limitations
Systematic review and meta-analysis	- Methodology applicable to RCT, observational studies and animal studies - Pooling results increases power to detect modest but clinically meaningful effects - Identify early evidence of harms vs. benefits of treatments and provide evidence-based recommendations	- Quality of summary result is dependent on the quality of the included studies - Often substantial between study heterogeneity - Not all available evidence is published and this may give biased results
Individual patient level data from completed trials	- Pooling data increases statistical power - Greater opportunity and flexibility to explore new research questions than traditional aggregate meta-analysis - RCT level quality control of source data with standardised, validated, monitored data points - Cost effective approach, saving resources and time	- Limited generalizability - Risk of bias of different RCT populations - Analyses restricted to available data - Inconsistency in tools used to assess outcomes may limit potential, this is a particular issue in studies of cognition
Individual participant level data from cohort studies	- Opportunity for large multicentre research platforms - ‘last word’ science - Strong epidemiological focus	- Limitations inherent to observational studies - Reverse causality - Confounding
Big data informatics	- Large data volume – cohort size, registries - Heterogeneity of data - multimodality - Potential for semi-automated data analyses	- Patient data confidentiality to be protected - Development of data sharing mechanisms - Complex computational methods and support required
Data linkage and use of routinely recorded data	- Resource-efficient - variety of differing data sources available, not solely limited to traditional health settings - Cross-sectional studies can turned into longitudinal, e.g. follow up a cohort of people with dementia for hospital admissions, death etc.	- Selection bias depending on type of data used (e.g. hospital admissions) - Quality of data might vary - Non-standardised outcome measures

## Methods

This paper is a result of a dynamic and iterative process. The lead authors first reviewed the published literature and identified key thematic areas. Organisations or research groups working in these areas were identified and invited to contribute. The consensus was further developed as a key topic area of the International Congress on Vascular Dementia in Ljubljana, Slovenia (16^th^ – 18^th^ October 2015). Comments were collated following a plenary session and open invitation workshop at the conference. Key stakeholders met for a round table meeting and finalised the content of the overview. Topic leads (MA, GDB, GM, TR, RS) drafted each specific section of the overview with synthesis and editing by ICVD data theme leads (FD, TQ). The draft was shared with other researchers and centres who expressed an interest, and subsequent discussion and revision continued until a final agreed text was reached.

## Big data

The “big data” concept is a hot topic in contemporary research but there is continuing debate over the meaning of the phrase [4]. According to the Gartner group definition, “big data” is characterized by the following properties:

- large data volume (“volume”)
- heterogeneity and disparity of data (“variety”)
- the speed with which data are being generated (“velocity”)
- quality and integrity of data (“veracity”)

The fourth “V” was added to illustrate the need for critical assessment of the quality of data:

Under the rubric of big data we can consider various information sources and various approaches to curation and analysis. Data can be “big” in terms of breadth (number of individuals, for example national data registries) or “big” in terms of depth (level of detail on each individual, for example sophisticated neuroimaging). Often data are “big” in both senses (for example a population registry with multimodal data such as UK Biobank).

Large data sets are being generated in traditional spheres of science and health but also in many other aspects of everyday life, internet usage; social media; shopping habits etc. The “omics” arena (a term used to describe the collection of scientific technologies that investigate the mechanisms of molecules and their interactions within a cell) is an example of a potentially transformative activity using large datasets. With the advent of Next Generation Sequencing (NGS) technologies, a new scale of data production has been reached: sequencing one person's genome produces approximately 4 terabytes of raw data output.

The landscape is evolving and the combined effect of technological advances (increasing capacity and decreasing cost of data storage) and healthcare systems change (increasing electronic recording of patient data) make for a rich environment to facilitate big data research in VaD. If we extrapolate what can be observed in immunology and cancer biology, we should prepare for a tsunami of data in dementia research in the near future. Entire patient cohorts will be fully sequenced at reasonable price and within days; we can expect very soon a high resolution representation of genome variation in large studies such as EPAD (European Prevention of Alzheimer’s Dementia), ADNI (for Alzheimer´s Disease) and PPMI (for Parkinson´s Disease).

At the same time, we see an urgent need to enhance the interpretability of “big data” based on the current state of knowledge. Technologies to extract and to represent essential knowledge are now mature enough to allow for the rapid construction of knowledge-based models for entire indication areas. Algorithms such as “reverse causal reasoning” [5] allow for a rapid analysis of whether a given data set represents the causal and correlative relationship patterns in a knowledge-based model. As a consequence, semi-automated data analysis will be possible at large scale and high throughput, matching essential requirements for big data processing and analysis in the future.

We can expect that, in the near future, data production at all levels - from the omics level to the clinical and population level - will increase at the same rate in dementia research as can be observed in other indication areas. The need for increased interoperability of data, and validation of data, will simultaneously increase, and substantial effort will be required to cope not only with the rapid growth of data volume, but also with the notorious lack of interoperability of data, information, and knowledge. We will see more ambitious mining scenarios in big data challenges in the future, and integrative modelling and mining approaches.

## Case-study: scientific and patent literature

These documents contain information in disparate formats: unstructured semantic data (text); structured data (tables); and graphically encoded information (chemical structures and their interactions). From the Big Data perspective, the literature data pools are incomplete and of varying quality. Only a fraction of the more recent biomedical peer reviewed literature offers unrestricted access to machine-readable full text. Patent documents, while freely available online, provide only scanned bitmaps and/or raw optical character recognition output in most cases, and their ontology does not readily integrate with the corresponding peer review literature [6].

These deficiencies notwithstanding, the corpus of published scientific information allows targeted as well as parameter-free data mining projects that could reveal not only unexpected risk factors and interactions that are relevant for VaD, but could also uncover drug effects and side effects that provide invaluable clues to the re-development of known drugs and drug candidates. If literature mining is combined with mining patient-level data from clinical trials and/or postmarketing side effect data reports [7], drug repurposing research has almost unlimited potential. It requires a multidimensional approach that is based on two fundamental facts:

- All disorders are driven along molecularly defined molecular pathways, which have interaction nodes that can be modulated, often in multiple ways; and
- All drugs are multifunctional.

Provided that the biological pathways and their interactions are understood, and that the pharmacological activities of the drug compounds are known, therapeutically meaningful new combinations can be identified using either exploratory or targeted (“drill-down”) algorithms, or most likely by their sequential application [8]. In VaD drug discovery, such efforts could be directed towards identifying known compounds that interfere with many critical points in the vicious cycle of cerebral small vessel disease, reduced blood flow and metabolism and neurological damage.

## Systematic review and meta-analysis

An obvious, but to date underused, resource for VaD research comes from synthesis of existing trial data. Historically, the research community has been guilty of pursuing research in areas where sufficient evidence is available [9]. This is both inefficient and unethical and a comprehensive review is a crucial first step in research.

Systematic review of the literature could be used to inform VaD research in a number of ways:

- Answering important research questions without needing to do further primary research
- Identifying ‘knowledge gaps’ and so setting the research agenda
- Informing sample size calculations for larger trials
- Identifying early evidence of harms and benefits of treatments

For the field of VaD research, scoping reviews by Cochrane and other groups, suggest that there is a fairly limited original research base on interventions for VaD. Thus it seems unlikely that systematic review will provide definitive answers, but comprehensive and critical synthesis of the available literature could assist in the choice of interventions for assessment and in the design of VaD trials.

Systematic review is not limited to human research. Reviews of animal studies can inform the translational medicine pathway. For example, a systematic review of antidepressants in animal models of stroke showed reduction in infarct size and improvement in neurobehavioural scores [10], consistent with the apparently beneficial effects of selective serotonin reuptake inhibitors on recovery after stroke [11] and supported methodologically robust ongoing clinical trials testing fluoxetine for stroke recovery [12]. Groups such as CAMARADES (Collaborative Approach to Meta-Analysis and Review of Animal Data from Experimental Studies) have raised standards in systematic review of animal studies.

Systematic review methodologies are also available to facilitate reviews of observational studies, diagnostic test accuracy and qualitative original research. For some of these areas, guidance is emerging on best practice in conduct, reporting and quality assessment. These materials have had a general dementia focus and will be just as applicable to VaD as to other dementia syndromes [13–15].

A systematic review should be considered as an experiment. There should be a clearly defined research question and methods which are described in advance in a protocol. Systematic reviews should have stated objectives with pre-defined eligibility criteria for studies, explicit, reproducible methodology, comprehensive search strategies that attempt to identify all studies (including unpublished studies), assessment of the validity of the findings of the included studies (including risk of bias and generalisability), systematic presentation, and synthesis of characteristics and findings and if possible, a meta-analysis.

## Case study – the Cochrane stroke and dementia groups

Cochrane (formerly the Cochrane Collaboration) is an international organisation of 37,000 contributors from over 130 countries (http://www.cochrane.org). Cochrane gathers and summarizes the best evidence from research to help patients, clinicians and policy makers make informed choices about treatments. During the past 20 years, Cochrane has transformed the way health decisions are made.

Cochrane groups include healthcare subject-related review groups, thematic networks, methodology groups and regional centres located all over the world. There is no Cochrane group exclusively for VaD research. The topic is most aligned with the Cochrane Stroke Group and the Cochrane Dementia and Cognitive Improvement Group (CDCIG). Both groups are exemplars of how Cochrane can produce clinically important outputs, shape the research agenda and develop new approaches to data.

The CDCIG (http://dementia.cochrane.org) has over 200 reviews and a comprehensive, open-access register of randomised controlled clinical trials or studies of diagnostic tests in dementia treatment, prevention and cognitive enhancement: ALOIS (http://www.medicine.ox.ac.uk/alois/). CDCIG reviews with a VaD focus are available [16] but numbers are modest in comparison to Alzheimer’s disease dementia. The Cochrane Stroke Group produces reviews on all aspects of stroke care. The group has produced several reviews on the management of cognitive deficits after stroke, an area that could be considered part of the VaD remit [17]. These reviews have demonstrated a paucity of trials, indicating that the management of post-stroke cognitive impairment is an important area for future research. The Cochrane Stroke Group hosts DORIS (The Database of Research in Stroke) which contains over 22,000 references to trials in an easy to search study-based form.

Both the CDCIG and Stroke groups would welcome expressions of interest to work on a synthesis of available data pertinent to VaD and related conditions.

## Individual-patient level data from completed trials

Completed RCTs offer a rich source of high quality, individual patient level data on demographics, clinical features, treatments and adverse events across a range of time points. Yet, following trial completion and publication, raw datasets often reside in industry or academic archives. If patient level data can be accessed and pooled from a number of trials then the statistical power and opportunity to explore new questions is increased substantially. For a field like VaD where we have limited original research, improving the value of the available datasets becomes even more important.

Re-use of anonymised RCT data has several benefits:

- RCT data are standardised, high quality, validated, and robustly monitored
- The reuse of existing RCT data is cost effective.
- Development of definitive studies can be expedited by using existing data, saving time and money. In contrast, prospective data collection on a similar population and subsequent analyses can add years to the research timeline.

The principal limitation of collating individual patient level data is that clinical trial datasets do not usually provide a representative sample. This is a particular problem in VaD and where trials are available they are often limited to a specific subgroup, for example mild cognitive impairment. Furthermore any analyses will be restricted to those data and endpoints that have been collected in trials. This can be problematic when trialists use a variety of assessments for an outcome, a particular issue in VaD research where a plethora of tools are used to measure cognition [18].

## Case study – the virtual international stroke trials archive (VISTA) and VISTA-cognition

The Virtual International Stroke Trials Archive (VISTA) [19] was developed with the aim of collating and providing access to clinical stroke trial data for novel analyses. The resource is home to more than 82,000 anonymised individual patients’ data and has facilitated more than 80 peer-reviewed publications on a range of topics. The VISTA resource has been used to pilot novel elements of RCT design, develop and validate prognostic tools and optimise endpoints for future RCTs [20–22].

The VISTA founding members recognised that post stroke cognitive impairment is a major issue but has been relatively under-studied [23] and so have created a resource to lodge data on prospective stroke studies with a neuropsychological focus (VISTA-Cognition). Established in summer 2015, the resource already holds data on 2,422 individuals across 8 studies, with commitments to contribute data from a further 6 large studies (http://www.vista.gla.ac.uk/index.php/vista-cognition). Following approval by the relevant Steering Committee, anonymised datasets are compiled and sent to the investigator for analyses. The first dedicated outputs from VISTA-Cognition are awaited but studies from the VISTA resource are already advancing our understanding of VaD [24].

## Completed and on-going cohort studies

Large-scale observational cohort studies have informed much of our understanding of VaD. Despite increasing financial investment in routinely collected data and their potential for linkage, investigator-led (field-based) cohort studies still have an important research role both for single- (‘discovery’ science) and multi-study analyses (‘definitive' science). Ongoing work is bringing together existing cohort studies with dementia ascertainment to create a platform for research as well as identifying existing cohorts that may not have a (vascular) dementia focus but could still contain data of relevance to VaD research. An example is Dementia Platforms UK (DPUK) a collaboration between academic centres and industry, established by the Medical Research Council, with a remit of sharing data and catalysing translational dementia research.

An ideal cohort substrate for VaD research would be a longitudinal, population-based study with repeated measures of cognition, clinical (including detailed vascular assessment) and sociodemographic data. A recent Joint Programme for Neurodegenerative Disease (JPND) research consortium has identified over 90 cohorts (600,000 participants) with data that could be used to explore the vascular contribution to cognitive decline [25]. Even with relatively lengthy follow-up and detailed cognitive examination the number of incident VaD diagnosis in population cohorts is likely to be modest and statistical methods for modelling cognitive decline may need to be employed [26].

The approach of pooling raw observational data across studies, with some refinements, was originally used in advancing our understanding of the importance of blood pressure, cholesterol, and weight for chronic disease risk [27, 28]. Although, as yet, these cohorts have not been used in a VaD context, more recently, this work has been extended to focus on psychological risk markers. This work has found that even moderate levels of distress are associated with elevated mortality [29]; personality types is unrelated to cancer risk [30] and reduced height, a marker of early life environmental insults, and socioeconomic disadvantage is linked to an elevated risk of all dementias combined [31]. This pooling of cohort studies is limited by the perennial shortcomings of observational studies themselves; chiefly, confounding and reverse causality.

## Case study – the UCL Scottish Health Survey and the Health Survey for England collaboration

The Health and Social Surveys Research Group within the Department of Epidemiology and Public Health at University College London has, for over 20 years, been responsible for designing, implementing, and curating data from the Scottish Health Survey and the Health Survey for England. These are a series of annual, independent, geographically representative health examinations of adults from the general population living in private households in Scotland and England. Crucially for the purposes of individual-participant meta-analysis, the methodology of these studies is near-identical [32, 33]. The prospective element to the study has been provided by the linkage of consenting study members to the National Health Service mortality register. This process of prospective, repeated phenotyping of a defined population with opportunity to link to other health records is urgently required to progress the VaD research field.

## Informatics and electronic patient records

The adoption of electronic patient records (EPRs) in routine clinical care is generating hitherto unseen volumes of data both in scale (case numbers) and depth (quantity of information on individual cases) [34]. The use of EPRs for research is in its infancy and its focus has been an overly narrow one on technical solutions; however, a more pressing need is to develop expertise in data use, so that the data resources are not wasted on questions for which they are not best suited. There is mileage in an EPR based approach to VaD but the best use of these data requires some consideration.

While there may be some utility for EPR data in VaD risk factor studies, these questions are often better answered using traditional research designs, which are less impeded by missing and/or biased data. Furthermore, a risk factor whose rarity requires the very large samples offered by EPRs for its detection may be of little clinical relevance, and rare but important risk factors may best investigated with other experimental designs. Where EPR datasets are strongest is in allowing naturalistic follow-up of sizeable patient cohorts receiving routine interventions. Therefore they would be particularly valuable for evaluating VaD disease course (e.g. who gets better, who gets worse, or cognitive trajectories post diagnosis) and determinants of response to intervention (e.g. who benefits most, who is most vulnerable to adverse effects) although confounding by indication needs to be carefully considered. Indeed, in these scenarios, EPR datasets are often the platform of choice, because bespoke clinical cohorts (i.e. those specifically recruited and examined) are limited in size and generalisability, and combined RCT samples even more so.

Having been a resource for some time in primary care, large EPR datasets have begun to accumulate in specialist services, including those providing dementia care [35]. Applications relevant to VaD have included an evaluation of relative response to acetyl cholinesterase inhibitor treatment [36] and mortality associated with antipsychotic use [37]. Natural language processing techniques offer the potential for expanding the depth of data for analysis through ‘unlocking’ information which is traditionally recorded in text rather than structured fields in the record. [38] However, arguably the most pressing challenge for EPR-derived research in dementia is the diversity of services providing care. For example, a typical ‘patient journey’ might involve a detailed assessment for diagnosis in specialist care, and useful cross-sectional data, but then discharge is relatively rapid back to primary care, followed by sporadic contacts with specialist care (e.g. for behavioural symptom management) and acute care (for dementia-related and/or incidental hospitalisations). If the main application of EPR research, as previously argued, is to evaluate the course and progression of a disorder, this is limited in dementia because only specialist care records are likely to contain direct evaluations of dementia status, such as measures of cognitive function.

So what are the potential solutions? Increased dementia-specific routine data might accrue in future if a clinical rationale emerges for recording these; however, this is currently not a foreseeable scenario. Proxy measures of disease progression might also be derived from multiple data sources, although this requires comprehensive data linkage across primary, acute, and specialist care, as well as potentially social care. Wearable or home-based devices might be used to track progression, although remain experimental and, again, need to demonstrate clinical applicability. Finally, shared records systems allowing patient and carer input might provide novel opportunities for informative feedback on ‘real world’ outcomes.

In Sweden the development of quality registries to improve quality of care for different disorders has been very successful and has led to clear improvements in care. EPR data in Sweden are not developed enough to extract research data, and throughout the country many different EPR systems are used. The quality registries collect data from the health care system and outcomes are often quality indicators developed by national guidelines for diseases in question. The Swedish Dementia registry, SveDem, registers dementia disorders at the time of diagnosis with a yearly follow-up. The registry has a national coverage of about 40% of all incident dementia cases in Sweden [39]. Using personal identification numbers, SveDem can be linked to other registries such as the national patient registry, the national drug prescription registry, deaths collated to national population registries, and other quality registries or biomarker databases [40, 41]. The sheer size of the registry (>65,000 patients with dementia) has made it possible, for example, to study mortality between different dementia disorders [42].

## Data linkage and use of routinely recorded data

“Each person in the world creates a Book of Life. This Book starts with birth and ends with death. Its pages are made up of the records of the principal events in life. Record linkage is the name given to the process of assembling the pages of this Book, into a volume. The Book has many pages for some and is but a few pages in length for others.” [43].

Assembling an individual’s contacts with health services – and often non-health institutions such as social care, education, or criminal justice – is a resource-efficient way to collate a large volume of data which have already been recorded for other purposes. The power of this methodology is that it can turn cross-sectional studies into longitudinal ones, whether data linkage is used to follow up a cohort of people with dementia [31, 44], or whether it is used to identify incident dementia in a general population sample [45]. This approach is particularly attractive for a condition such as VaD, where an individual is likely to be assessed and treated by a variety of health and social care agencies with greater and lesser involvement of differing disciplines as the disease progresses.

Using already collected data allows for large scale studies at a fraction of the cost and time that would be required to run a prospective outcomes study, while having data available across whole populations allows for investigation of uncommon conditions. The greater use and sophistication of information technology in healthcare allows further opportunity to use routine clinical data for research at a national or even international level.

There are a number of limitations to this methodology which must be borne in mind, particularly when data linkage is used to identify people with dementia. If hospital admission records are used, there is immediate selection bias because not everyone will be admitted to hospital. Once admitted to hospital, dementia only seems to be recorded on discharge about half the time [45]. However, individuals who are admitted multiple times stand more chance of their diagnosis being recorded on at least one occasion. Again, these issues are even more problematic if the focus is a specific dementia subtype such as VaD, as often this level of granularity of diagnosis is not routinely captured. Multiple admissions are more common in those with dementia diagnoses. Furthermore, one cannot often infer the date of diagnosis, other than to state that dementia occurrence and diagnosis must have occurred before the first record mentioning dementia. Perhaps more importantly one cannot infer the timing of diagnosis, for example an early diagnosis made in primary care versus a diagnosis of late stage dementia when a person is resident in a care-home. Timing of dementia diagnosis is crucial to understanding potential direction of causation in studies looking at risk factors for dementia. If no unique identifier is available for each individual then probabilistic matching algorithms with an arbitrary threshold must be used to ensure that all the records associated with that person truly refer to them.

Death records have previously been thought to be inadequate for use in epidemiological studies [46] but rates of reporting are improving. Importantly, because people with dementia often die of something else, it is essential to look for ‘any mention’ of dementia on the death certificate, rather than merely looking at the underlying cause of death. Thus, in a tertiary referral memory clinic sample, 72% of people with robustly diagnosed dementia had their diagnosis correctly recorded on their death certificate [44]. However, the codes recorded are often non-specific dementia rather than diagnostic subtype. Thus, all-cause dementia ascertained from data linkage is likely to be a reasonably robust outcome, as are rare subtypes such as Fronto Temporal Dementia which are likely to be correct if they are recorded. In contrast, more common subtypes such as Alzheimer’s dementia and VaD are probably less robustly identified from routinely recorded data at the moment. This issue may be less important than previously thought, as the aetiological classification of dementia is evolving and concepts of pure Alzheimer's Disease and pure VaD are now considered less useful in older adults with dementia. One specific area in which this technique might have utility is in post-stroke dementia since acute stroke is arguably better identified, with high accuracy and clear times of diagnosis. Thus, a cohort of stroke survivors could be identified to follow up or, possibly, people who have dementia recorded following some record of a stroke could be found.

## Case-study – using routinely recorded data in Scotland

Scotland, along with certain other European countries, is well placed for exploiting data linkage as a means to progress VaD research. Everyone born or living in Scotland is issued with a unique identifier (the 10-digit Community Health Index number [CHI]). The CHI number is issued to label all encounters within NHS Scotland and is also used for national datasets such as mortality (death certification). National resources that also use CHI labelling include the NHS Central Register which notifies deaths with up to six causes of death recorded and the Scottish Morbidity Records (SMR). SMR includes various domains, for example records of discharges from acute hospitals in Scotland (SMR01), mental health hospitals in Scotland (SMR04) and Scottish cancer registrations (SMR06). Each discharge record contains up to 6 diagnoses, coded using International Classification of Diseases codes. Broader aspects of health and social care are being recorded with CHI labelling, for example admission to care-home and, in certain parts of Scotland, use of home-care services. The healthcare system in Scotland, where there is little use of exclusive private healthcare and almost all medical encounters are within the NHS, ensures comprehensive population coverage that is CHI labelled. In theory, using CHI linkage offers the potential to create a pan-national cohort. However, the lack of a specific electronic patient record for cognitive impairment and dementia undermines the capacity to utilise the otherwise excellent data in Scotland.

## Standardised outcome measurement

A recurring theme in our discussion of data driven VaD research is around the validity of the data availability when there is significant variation in the types and definitions used for VaD outcome measurement. The International Consortium for Health Outcome Measurement (ICHOM) is a not-for-profit organization that was co-founded by leaders from The Karolinska Institute, The Boston Consulting Group, and Harvard Business School and is uniquely grounded in a solid theoretical framework: value based health care [47, 48]. ICHOM’s goal is to develop Standard Sets of outcomes, because a standardised approach aids comparisons of outcomes across cultures, countries and healthcare systems. ICHOM believe this approach allows teams of health professionals to learn from one another, and enables institutions to use data to benchmark against each other, foster dialogue around variations in outcomes and learn from the best. ICHOM brings together leading healthcare professionals, registry leaders, outcome measurement experts and patient advocates to develop globally agreed Standard Sets of outcome measures that matter most to patients, for the world’s most burdensome medical conditions. The ICHOM methodology for creating Standard Sets is well-established, and brings together a literature review, review of existing registries, modified Delphi consensus processes, patient focus groups, an open consultation, and an expert working group that includes patients at every stage. ICHOM have recently developed and published the Dementia Standard Set, which is being piloted across institutions to identify the ‘best-in-class’ outcomes which can then drive health care improvements (http://www.ichom.org/medical-conditions/dementia/).

## Conclusion

This review demonstrates approaches to data that may assist in elucidating the causes and determining the treatment of VaD (an area of significant unmet need). “Big data” approaches can either use routinely collected clinical data (where clear challenges exist in ensuring complete capture of all health care records) or can be adopted to mine other available data to devise and answer novel questions. A caution with all these approaches is that they are reliant on the original data and study design. Big data does not necessarily mean better data.

There has been recent major financial investment by research agencies in the use of new approaches to data and considerable excitement in the scientific community as a result It has been said that the utility of new approaches to data will be most apparent in those areas where traditional approaches to clinical research have still to deliver effective treatments. In this regard the VaD research community should embrace the new opportunities and make full use of all the available resources.

**Table:** 

Box 1 Questions and future research directions around data driven VaD research	
1. Systematic review of VaD research: Are search strategies sufficiently sensitive to ensure all VaD research is returned? VaD is a condition with confusing terminology and various synonyms that include imaging descriptors and eponymous syndromes. It is possible that generic dementia search filters may not capture potentially relevant papers. Research required: The ideal would be a harmonised approached to indexing but in the meantime, validation of VaD search filters and refinement are needed. These issues are equally pertinent to outcomes of interest in VaD research such as institutionalization. 2. Pooling individual participant level data from existing trials in VaD: Do the data collected in historical VaD or stroke trials include the aspects of greatest relevance to contemporary VaD research? As the VaD research agenda evolves certain research questions are answered and new fields of enquiry emerge. Secondary analysis of existing data is constrained by the factors measured by the original investigators. Research required: We need original research that describes the data that are most important to key stake holders. Consensus meetings such as ICVD allow the research community to design studies that include data that may be used for future secondary analyses. Potentially more important is describing what are important outcomes for the trial participants themselves. Datasets often collect mortality and vascular morbidity but functional outcomes and quality of life are less commonly recorded. 3. Using data from existing cohorts to understand VaD: Which cohort populations are best suited for VaD research? We are developing large community cohorts such as UK Biobank, but will a cohort of younger participants have sufficient VaD cases to allow robust estimates? Specialist cohorts with a VaD focus will be more limited in size and may be too late in the VaD process to determine potentially modifiable risk factors. Research required: Bespoke cohorts may be required. Stroke cohorts may have particular value, as stroke is associated with high incidence of cognitive impairment and could be seen as an “enriched sample” for investigations around vascular cognitive impairment. 4. Electronic Health Records and VaD: How valid are recorded diagnoses of dementia and dementia subtypes in routine (non-specialist) health records? Is dementia recorded where it is a comorbidity and not the principal reason for healthcare consultation. If dementia is recorded is a dementia subtype recorded. Research required: We need comparisons of routinely recorded data against well phenotyped populations. Large scale research projects such as UK Biobank should allow for these analyses.	

## Abbreviations

ADNI: Alzheimer’s disease neuroimaging initiative
CAMARADES: Collaborative approach to meta-analysis and review of animal data from experimental studies
CDCIG: Cochrane Dementia and Cognitive Improvement Group
CHI: Community health index
DORIS: The database of research in stroke
DPUK: Dementia Platforms United Kingdom
EPAD: European Prevention of Alzheimer’s Dementia
EPR: Electronic patient record
FTD: Fronto temporal dementia
ICHOM: International consortium for health outcome measurement
JPND: Joint programme for neurodegenerative disease
NGS: Next generation sequencing
NHS: National health service
PPMI: Parkinson´s progression markers initiative
RCT: Randomized controlled trial
SHS: Scottish Health Survey
SMR: Scottish morbidity records
SveDem: Swedish Dementia registry
UCL: University College London
VaD: Vascular Dementia
VISTA: Virtual international stroke trials archive
